# New Genotypes of *Enterocytozoon bieneusi* Isolated from Sika Deer and Red Deer in China

**DOI:** 10.3389/fmicb.2017.00879

**Published:** 2017-05-18

**Authors:** Jianying Huang, Zhenjie Zhang, Yong Yang, Rongjun Wang, Jinfeng Zhao, Fuchun Jian, Changshen Ning, Longxian Zhang

**Affiliations:** ^1^College of Animal Science and Veterinary Medicine, Henan Agricultural UniversityZhengzhou, China; ^2^Wu Xi Medical School, Jiangnan UniversityWuxi, China; ^3^State Key Laboratory for Molecular Biology of Special Economic Animals, Institute of Special Economic Animal and Plant Science, Chinese Academy of Agricultural SciencesChangchun, China

**Keywords:** *Enterocytozoon bieneusi*, genotyping, internal transcribed spacer, deer, China

## Abstract

To examine the occurrence and genotype distribution of *Enterocytozoon bieneusi* in cervids, 615 fecal samples were collected from red deer (*Cervus elaphus*) and sika deer (*Cervus nippon*) on 10 different farms in Henan and Jilin Province. *Enterocytozoon bieneusi* was identified and genotyped with a nested PCR analysis of the internal transcribed spacer (ITS) region of the rRNA genes, showing an average infection rate of 35.9% (221/615). In this study, 25 ITS genotypes were identified including seven known genotypes (BEB6, EbpC, EbpA, D, HLJDI, HLJD-IV, and COS-I) and 18 novel genotypes (designated JLD-I to JLD-XIV, HND-I to HND-IV). Among these, BEB6 (131/221, 59.3%) was the predominant genotype (*P* < 0.01), followed by HLJDI (18/221, 8.1%) and JLD-VIII (16/221, 7.2%). BEB6 has recently been detected in humans and nonhuman primates in China. The phylogenetic analysis showed that BEB6, HLJDI, HLJD-IV, COS-I, and 10 novel genotypes (JLD-VII to JLD-XIV, HND-III to HND-IV) clustered in group 2. Genotype D, EbpC, and EbpA, known to cause human microsporidiosis worldwide, clustered in group 1, the members of which have zoonotic potential, together with eight novel genotypes (JLD-I to JLD-VI, HND-I to HND-II). Therefore, deer may play a role in the transmission of *E. bieneusi* to humans.

## Introduction

*Enterocytozoon bieneusi* is an obligate intracellular Fungi that infects a wide range of hosts, including humans, nonhuman primates, pigs, cattle, horses, llamas, kudus, dogs, cats, foxes, raccoons, otters, ermines, bears, deer, guinea pigs, beavers, rabbits, muskrats, falcons, snakes, wild rodents, and other birds, as well as environmental samples (Santín and Fayer, 2011; Guo et al., 2014; Karim et al., 2014b,c; Zhao et al., 2014; Corradi, 2015; Zhang et al., 2015). The pathogen, which is responsible for more than 90% of human microsporidiosis, usually invades the epithelial cells of the small intestine, causing chronic diarrhea, malabsorption, and wasting syndrome in immunocompromised individuals, including patients with acquired immunodeficiency syndrome (AIDS) (Didier and Weiss, 2006). *Enterocytozoon bieneusi* infection in immunocompetent individuals has also been reported (Matos et al., 2012).

Based on a sequence analysis of the internal transcribed spacer (ITS) region of the ribosomal RNA (rRNA) genes, over 200 genotypes of *E. bieneusi* have been identified in different hosts and water samples, among which at least 60 genotypes have been detected in humans (Yang et al., 2014; Zhao et al., 2015b). In a phylogenetic analysis, all *E. bieneusi* ITS genotypes were divided into at least nine distinct genetic clusters (groups 1–8 and the outlier in dogs) (Karim et al., 2015). Group 1, also known as the zoonotic group, is responsible for most human *E. bieneusi* infections, and contains the vast majority of genotypes from various animal hosts (Fayer and Santin-Duran, 2014). Groups 2–8 and the outlier chiefly consist of genotypes that are host-adapted or found in wastewater (Guo et al., 2014; Karim et al., 2014b).

As one of the centers of cervid evolution in China, the total number accounts for more than 40% of the world deer population. Deer and their products are of high economic value and deer farming has become an important component of China's economic animal breeding industries. Sika deer (*Cervus nippon*) and red deer (*C. elaphus*) are the two most common deer species in China. Velvet antlers, used in traditional Chinese medicine, are one of the main products from sika deer and there are approximately 550,000 sika deer in China at present (Li et al., 2013).

Several studies about *E. bieneusi* infections have been conducted on white-tailed deer (*Odocoileus virginianus*), sika deer (*Cervus nippon*), red deer (*Cervus elaphus*), and Pere David's deer (*Elaphurus davidianus*) in the last 3 years (Guo et al., 2014; Zhao et al., 2014; Santin and Fayer, 2015; Zhang et al., 2015, 2016). In these studies, genotypes I, J,WL4, WL18, WL19, LW1, and DeerEb1 to DeerEb13 were detected in the USA, and genotypes BEB6, type IV, EbpC, EbpA, J, COS-I, COS-II, CHN-DC1, KIN-1, JLD-1 to JLD-3, and HLJD-I to HLJD-V were found in China (Table 1). Nevertheless, informations concerning *E. bieneusi* infections in cervids in the world are still limited. The aim of this study were to determine the prevalence and genetic characterization of *E. bieneusi* infections in cervids in Henan and Jilin Provinces.

**Table 1:** **Distribution of *Enterocytozoon bieneusi* genotypes in deer**.

**Cervid species**	**Locations**	**Infection rate**	**Genotype(s)**	**References**
White-tailed deer	New York, USA	12.2% (6/49)	WL18 (2), WL19 (2), WL4 (2)	Guo et al., 2014
White-tailed deer	Maryland, USA	32.5% (26/80)	WL4 (11), I (7), J (1), LW1 (1), DeerEb1-DeerEb13 (one each)	Santin and Fayer, 2015
Sika deer	Heilongjiang and Jilin Province, China	32.6% (28/86)	BEB6 (20), HLJD-I (1), HLJDII (1), HLJD-III (1), HLJD-IV (1), HLJD-V (4)	Zhao et al., 2014
Red deer	HeilongjiangProvince, China	20.0% (1/5)	HLJD-V (1)	Zhao et al., 2014
Pere David's deer	Henan Province, China	34.0% (16/47)	type IV (4), EbpC (4), EbpA (4), BEB6 (2), COS-I (1), COS-II (1)	Zhang et al., 2015
Sika deer	Jilin Province, China	7.1% (23/326)	J (11), BEB6 (4), EbpC (1), CHN-DC1 (1), KIN-1 (1), JLD-1(2), JLD-2 (2), JLD-3 (1)	Zhang et al., 2016
Sika deer	Jilin and Henan Province, China	35.9% (215/599)	BEB6 (129), HLJDI (18), EbpC (3), HLJD-IV (2), COS-I (1), EbpA (1), D (1), JLD-I (7), JLD-II (5), HND-I (4), JLD-III (2), HND-II (1), JLD-IV (3), JLD-V (2), JLD-VI (5), HND-III (1), JLD-VII (1), JLD-VIII (16), JLD-IX (1), JLD-X (1), HND-IV (1), JLD-XI (2), JLD-XII (1),JLD-XIV (7)	This study
Red deer	Jilin Province, China	37.5% (6/16)	BEB6 (2), JLD-IV (3), JLD-XIII (1)	This study

## Materials and methods

### Ethics statement

This study was performed strictly according to the recommendations of the Guide for the Care and Use of Laboratory Animals of the Ministry of Health, China. The research protocol was reviewed and approved by the Research Ethics Committee of Henan Agricultural University. Permission was obtained from the farm owners before the fecal samples were collected.

### Sample collection and examination

A total of 615 samples were collected between April 2014 and August 2014 on 10 farms in Henan and Jilin Provinces, from 599 sika deer and 16 red deer. These animals, which were in shed-feeding, were horsed in separate breeding houses according to different deer species and age groups. Approximately 30–50 g of fresh fecal sample was collected from each deer immediately after its defecation onto the ground, using a sterile disposal latex glove, and was then placed individually into a disposable plastic bag. No obvious clinical signs were observed in the sampled animals. There was a wide age distribution, ranging from 1 month to 15 years. All fecal specimens were stored in 2.5% potassium dichromate solution at 4°C until processing.

### DNA extraction

The fecal specimens were washed three times in distilled water with centrifugation at 3,000 × g for 10 min to remove the potassium dichromate. DNA was extracted from 200 mg of each fecal specimen using the E.Z.N.A. Stool DNA Kit (Omega Biotek Inc., Norcross, USA), according to the manufacturer's instructions. The extracted DNA was stored at −20°C.

### PCR amplification and sequence analysis

A nested PCR targeting a ~392-bp fragment of the ITS rRNA sequences was used to determine the genotypes of *E. bieneusi*. The primers were EBITS3 (5′-GGT CAT AGG GAT GAA GAG-3′) and EBITS4 (5′-TTC GAG TTC TTT CGC GCT C-3′) for the primary PCR, and EBITS1 (5′-GCT CTG AAT ATC TAT GGC T-3′) and EBITS2.4 (5′-ATC GCC GAC GGA TCC AAG TG-3′) for the secondary PCR. The reaction conditions were described in a previous study (Buckholt et al., 2002). KOD-Plus DNA polymerase (Toyobo Co., Ltd, Osaka, Japan) was used for PCR amplification. All PCR amplicons were sequenced on an ABI Prism™ 3730 XL DNA Analyzer using the BigDye Terminator v3.1 Cycle Sequencing Kit (Applied Biosystems, Foster, CA, USA). The sequencing accuracy was confirmed with two-directional sequencing. The sequences were identified by their alignment with reference sequences downloaded from GenBank (http://www.ncbi.nlm.nih.gov), using the MEGA 6.0 software (http://www.megasoftware.net). Phylogenetic trees were constructed with the neighbor-joining method (Kimura two-parameter model) in the MEGA 6.0 software, and a bootstrap analysis with 1,000 replicates was used to assess the reliability of the trees. Representatives of all the nucleotide sequences generated in this study have been deposited in GenBank under accession numbers KX383614-KX383647.

### Statistical analysis

A χ^2^ test was used to compare the *E. bieneusi* infection rates. Differences were considered significant at *P* < 0.05.

## Results

### Prevalence of *E. bieneusi*

In this study, the overall prevalence of *E. bieneusi* in cervids was 35.9% (221/615), with infection rates of 35.9% (215/599) in sika deer and 37.5% (6/16) in red deer (*P* > 0.05). All 10 farms were positive for *E. bieneusi*, with infection rates ranging from 30.0 to 53.9% (Table 2). The prevalence of *E. bieneusi* in Jilin and Henan Provinces was 37.2% (172/463) and 32.2% (49/152), respectively, and the difference was not significant (*P* > 0.05). However, the prevalence of *E. bieneusi* was significantly higher in males (42.1%, 126/299) than in females (30.1%, 95/316) (*P* < 0.01). The prevalence of *E. bieneusi* was higher in the 6–12 month age group (90/158, 57.0%) than that in the <6 month (29.0%, 9/31) and > 1 year age groups (28.7%, 122/426) (*P* < 0.01) (Table 3).

**Table 2:** **Infection status of *Enterocytozoon bieneusi* in deer**.

**Province**	**Farm**	**No. of positive/no.of examined**	**Genotypes (n)**
Jilin	Yutan-A	14/29 (48.3%)	BEB6 (9), JLD-VIII (4), JLD-IX (1)
	Yutan-B	35/65 (53.9%)	BEB6 (22), HLJDI (2), JLD-I (2), JLD-V (2), JLD-VIII (5), JLD-XI (1), JLD-XIV (1)
	Shuangyang-A	22/52 (42.3%)	BEB6 (17), JLD-II (1), JLD-III (1), JLD-IV (2), JLD-VI (1)
	Shuangyang-B	11/32 (34.4%)	BEB6 (3), HLJDI (2), JLD-I (2), JLD-IV (4)
	Zuojia-A	19/50 (38.0%)	BEB6 (14), HLJDI (1), EbpC (1), JLD-III (1), JLD-VII (1), JLD-XIII (1)
	Zuojia-B	4/13 (30.8%)	BEB6 (1), HLJD-IV (2), JLD-XII (1)
	Zuojia-C	36/120 (30.0%)	BEB6 (23), HLJDI (3), JLD-I (1), JLD-II (3), JLD-VIII (5), JLD-XIV (1)
	Tonghua	31/102 (30.4%)	BEB6 (25), JLD-I (2), JLD-VI (1), JLD-VIII (2), JLD-X (1)
Henan	Xinxian	17/56 (30.4%)	BEB6 (10), HLJDI (5), EbpC (1), HND-II (1)
	Qixian	32/96 (33.3%)	BEB6 (7), HLJDI (5), EbpC (1), COS-I (1), EbpA (1), D (1), JLD-II (1), HND-I (4), JLD-VI (3), HND-III (1), HND-IV (1), JLD-XI (1), JLD-XIV (5)
Total		221/615 (35.9%)	BEB6 (131), HLJDI (18), EbpC (3), HLJD-IV (2), COS-I (1), EbpA (1), D (1), JLD-I (7), JLD-II (5), HND-I (4), JLD-III (2), HND-II (1), JLD-IV (6), JLD-V (2), JLD-VI (5), HND-III (1), JLD-VII (1), JLD-VIII (16), JLD-IX (1), JLD-X (1), HND-IV (1), JLD-XI (2), JLD-XII (1), JLD-XIII (1), JLD-XIV (7)

**Table 3:** **Prevalence of *E. bieneusi* in deer by age and sex**.

**Characteristics**	**Infection rate (%)**	**No. of positive**	**No. of examined**
**AGE**			
<6 months	29.0	9	31
6–12 months	57.0	90	158
>1 year	28.6	122	426
**SEX**			
Male	42.1	126	299
Female	30.1	95	316

### Genetic characterization and genotype distribution of *E. bieneusi* in deer species

In total, 25 *E. bieneusi* ITS genotypes were detected in this study. They included seven known genotypes (BEB6, EbpC, EbpA, D, HLJDI, HLJD-IV, and COS-I) and 18 novel genotypes (JLD-I to JLD-XIV, HND-I to HND-IV) (Table 2). Among these genotypes, 34 polymorphic sites were observed within the 243 bp of the ITS gene sequence (Table 4). BEB6 (131/221, 59.3%) was the predominant genotype in all age groups (*P* < 0.01), and found in all 10 farms, followed by HLJDI (8.1%, 18/221) and JLD-VIII (7.2%, 16/221). All genotypes but JLD-XIII (24 genotypes) were found in sika deer, whereas only BEB6, JLD-IV and JLD-XIII were detected in red deer. At least three genotypes were observed on each individual farm, and 13 genotypes were found on Qixian farm, including known zoonotic genotypes D, EbpC, and EbpA.

**Table 4:** **Variations in the ITS nucleotide sequences among genotypes of *Enterocytozoon bieneusi* isolated from deer in this study**.

**Genotypes**	**Nucleotide at sequence position**																																	
	**2**	**10**	**18**	**31**	**33**	**45**	**58**	**74**	**76**	**81**	**90**	**93**	**95**	**113**	**117**	**129**	**131**	**136**	**137**	**141**	**143**	**147**	**158**	**168**	**178**	**179**	**185**	**187**	**188**	**189**	**190**	**191**	**194**	**196**
BEB6	C	A	A	A	A	A	T	C	C	C	G	T	G	T	T	A	C	G	T	T	A	A	A	G	A	C	G	T	G	G	A	T	A	A
D	C	G	G	G	G	G	G	A	C	C	G	C	G	C	T	G	G	G	C	T	A	G	T	G	G	T	T	G	G	A	T	G	G	A
EbpA	C	G	G	A	G	G	G	A	C	T	G	T	T	T	G	G	G	A	C	T	G	G	T	G	G	T	T	G	G	A	T	G	G	G
EbpC	C	G	G	G	G	G	G	A	C	C	G	T	G	T	G	G	G	G	C	C	A	G	T	G	G	T	T	G	G	A	T	G	G	A
COS-I	C	A	A	A	A	A	T	C	T	C	G	T	G	T	T	A	C	G	T	T	A	A	A	G	A	C	G	T	G	G	A	T	A	A
HLJDI	C	G	G	G	G	G	G	C	C	C	G	T	G	T	T	A	C	G	T	T	A	A	A	G	A	C	G	T	G	G	A	T	A	A
HLJD-IV	C	G	A	G	A	G	G	A	C	C	G	T	G	T	T	G	G	G	T	T	A	A	A	T	G	T	T	G	G	A	T	G	G	A
JLD-I	C	G	G	G	G	G	G	C	C	C	G	T	G	C	T	G	G	G	C	T	A	G	A	G	G	T	T	G	G	A	T	G	G	A
JLD-II	C	G	G	G	G	G	G	A	C	C	G	T	G	C	T	G	G	G	C	T	A	G	T	G	G	T	T	G	G	A	T	G	G	A
HND-I	C	G	G	G	G	G	G	A	C	C	G	T	G	C	G	G	G	G	C	T	A	G	T	T	G	T	T	G	G	A	T	G	G	A
JLD-III	C	G	G	G	G	G	G	A	C	C	G	C	G	C	T	G	G	G	C	T	A	G	T	T	G	T	T	G	G	A	T	G	G	A
HND-II	C	G	G	G	G	G	G	A	C	C	G	T	G	T	G	G	G	G	C	C	A	G	T	T	G	T	T	G	G	A	T	G	G	A
JLD-IV	C	G	G	G	G	G	G	A	C	C	G	T	G	T	G	G	G	G	C	C	A	G	A	G	G	T	T	G	G	A	T	G	G	A
JLD-V	C	G	G	G	G	G	G	A	C	C	G	T	G	T	G	G	G	G	C	C	A	G	T	T	A	C	G	T	G	G	A	T	A	A
JLD-VI	C	G	G	G	G	G	G	C	C	C	G	T	G	T	G	G	G	G	C	C	A	G	T	G	G	T	T	G	G	A	T	G	G	A
HND-III	C	G	G	G	G	G	G	A	C	T	G	T	G	T	G	A	C	G	T	T	A	A	A	G	A	C	G	T	G	G	A	T	A	A
JLD-VII	C	A	A	A	A	A	T	C	C	C	G	C	G	T	T	A	C	G	T	T	A	A	A	G	G	T	–	T	G	G	A	T	A	A
JLD-VIII	C	G	G	G	G	G	G	A	C	T	G	T	G	T	G	A	C	G	T	T	A	A	A	G	G	T	T	G	G	A	T	G	G	A
JLD-IX	C	A	A	A	A	A	T	C	C	C	G	T	G	T	T	A	C	G	T	T	A	A	A	G	A	C	G	T	A	G	A	T	A	A
JLD-X	C	A	A	A	A	A	T	C	C	C	T	T	G	T	T	A	C	G	T	T	A	A	A	G	A	C	G	T	G	G	A	T	A	A
HND-IV	T	A	A	A	A	A	T	C	C	C	G	T	G	T	T	A	C	G	T	T	A	A	A	G	A	C	G	T	G	G	A	T	A	A
JLD-XI	C	G	G	G	G	G	G	A	C	C	G	C	G	T	T	A	C	G	T	T	A	A	A	G	A	C	G	T	G	G	A	T	A	A
JLD-XII	C	A	A	A	A	A	T	C	C	C	G	T	G	T	T	A	C	G	T	T	A	A	T	G	G	T	T	G	G	A	T	G	G	A
JLD-XIII	C	A	A	A	A	A	G	A	C	C	G	T	G	T	T	A	C	G	T	T	A	A	A	G	A	C	G	T	G	G	A	T	A	A
JLD-XIV	C	G	G	G	G	A	T	C	C	C	G	T	G	T	T	A	C	G	T	T	A	A	A	G	A	C	G	T	G	G	A	T	A	A

*The bars denote nucleotide deletion at the position*.

### Phylogenetic analysis

**Figure 1:**
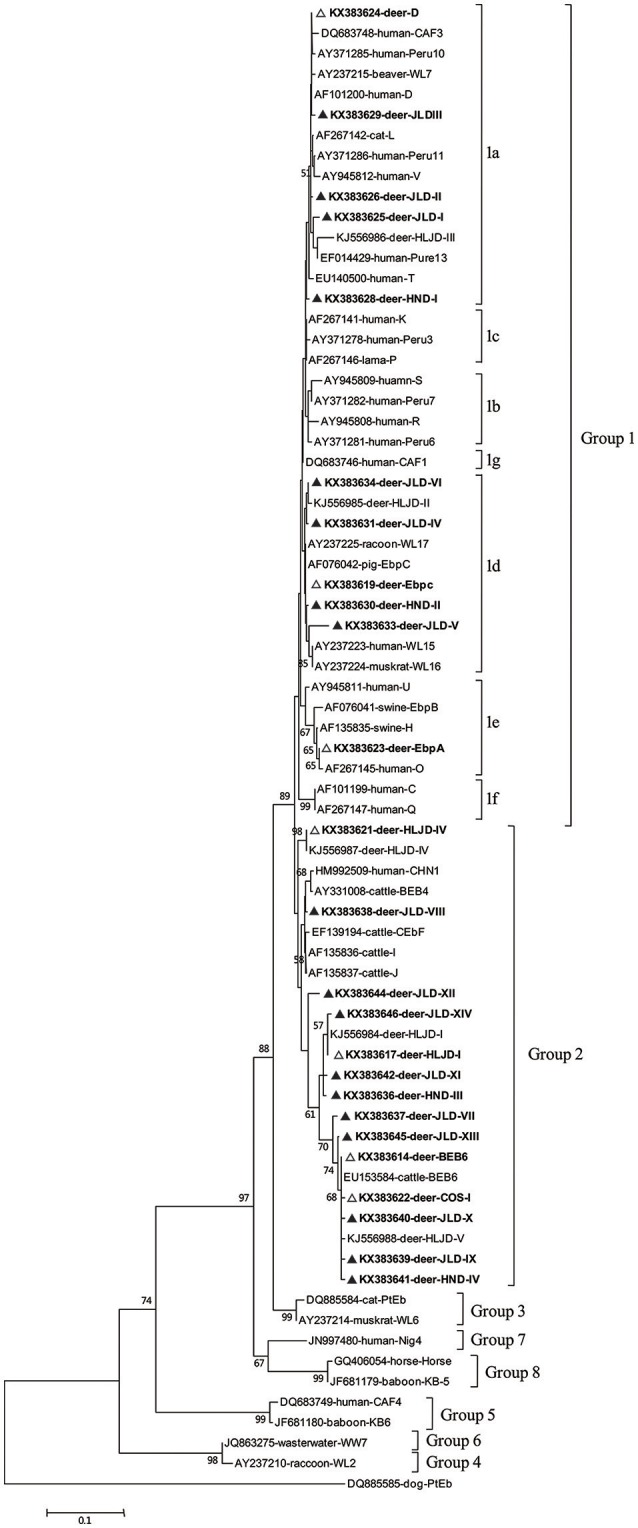
**Phylogenetic relationships of the *E. bieneusi* genotypes identified in this study and other reported genotypes**. The phylogeny was inferred with a neighbor-joining analysis of the ITS sequences based on distances calculated with the Kimura two-parameter model. Bootstrap values >50% from 1,000 replicates are shown on the nodes. The genotypes detected in this study are shown with triangles; known genotypes observed in this study are marked with open triangles and novel genotypes are indicated by filled triangles.

A phylogenetic analysis of the ITS sequences of all the *E. bieneusi* genotypes detected here and reference genotypes published previously indicated that 11 of the 25 genotypes detected in this study belonged to zoonotic group 1: genotypes D, HND-I, and JLD-I to JLDIII in subgroup 1a; genotypes EbpC, HND-II, and JLD-IV to JLD-VI in subgroup 1d; and genotype EbpA in subgroup 1e. The remaining 14 genotypes (BEB6, HLJDI, HLJD-IV, COS-I, JLD-VII to JLD-XIV, and HND-III to HND-IV) all clustered in group 2, the so-called “cattle-specific” group (Figure 1).

## Discussion

In this study, 35.9% of 615 deer were infected with *E. bieneusi*, demonstrating the common occurrence of this pathogen in cervids in China. These results are consistent with those published from Maryland, USA, in white-tailed deer (*Odocoileus virginianus*) (32.5%) (Santin and Fayer, 2015), and from Heilongjiang and Jilin Provinces, China, in sika deer and red deer (31.9%) (Zhao et al., 2014); and Henan Province, China, in Pere David's deer (*Elaphurus davidianus*) (34.0%) (Zhang et al., 2015), but in contrast with those from New York, USA, in white-tailed deer (12.2%) (Guo et al., 2014), and from Jilin Province, China, in sika deer (7.1%) (Zhang et al., 2016). Generally speaking, the infection rates in different studies are affected by many factors, including age distributions, specimen sizes, management systems, population density, health status of the hosts, and the sensitivity and specificity of the detection methods used, so it is difficult to explain the actual discrepancies in the prevalence of *E. bieneusi*.

There was little information on the molecular epidemiology of *E. bieneusi* in deer until 2014. Several recent studies conducting on white-tailed deer, sika deer, red deer, and Pere David's deer have identified Genotypes I, J,WL4, WL18, WL19, LW1, DeerEb1 to DeerEb13 in the USA and genotypes BEB6, type IV, EbpC, EbpA, J, COS-I, COS-II, CHN-DC1, KIN-1, JLD-1 to JLD-3, HLJD-I to HLJD-V in China (Guo et al., 2014; Zhao et al., 2014; Santin and Fayer, 2015; Zhang et al., 2015, 2016). In the present study, 25 genotypes (seven known and 18 novel) were identified with an analysis of the ITS sequences of 221 *E. bieneusi* isolates. Among these genotypes, BEB6 occurred in the highest percentage of *E. bieneusi*-positive specimens (59.3%, 131/221) and displayed the widest distribution across farms, which is consistent with the results of Zhao et al. (2014), who reported that BEB6 accounted for 69.0% of *E. bieneusi* isolates. This genotype appears to be common in ruminants, including cattle, sheep, goats, golden takins, deer, and alpacas (Li et al., 2014; Zhao et al., 2014; Zhao G.-H. et al., 2015; Ye et al., 2015; Shi et al., 2016). BEB6 has also been found in humans, rhesus macaques, cats, horses, and wastewater in China (Li et al., 2012; Wang et al., 2013a; Karim et al., 2014a,b; Qi et al., 2016). So far, deer infected with *E. bieneusi* genotype BEB6 have only been found in China. In the USA, genotypes WL18, WL4, and I seem predominant (Guo et al., 2014; Santin and Fayer, 2015). The absolute predominance of genotype BEB6 in this and other studies and the fact that BEB6 has been detected in humans indicate that systematic molecular epidemiological investigations of BEB6 in animals and humans are required.

Genotype D, considered the most common genotype in human microsporidiosis caused by *E. bieneusi*, has frequently been detected in both HIV-positive patients and HIV-negative individuals in America, Europe, Africa, and Asia, including Shanghai City and Henan Province, China (Akinbo et al., 2013; Wang et al., 2013a,b). This genotype appears to have a wide host range, including livestock (cattle, horses, pigs, and rabbits), pets (cats and dogs), wildlife (beavers, falcons, foxes, mice, muskrats, nonhuman primates, otters, raccoons, golden takins, and wild boars), and birds (pigeons) (Santín and Fayer, 2011; Fiuza et al., 2015; Yang et al., 2015), and also occurs in urban wastewater (Li et al., 2012) and drinking water (Guo et al., 2014). In this study, genotype D was found in sika deer for the first time, indicating that genotype D might have a more extensive range of reservoir hosts than expected. EbpA and EbpC are another two prevalent genotypes found in humans and many animal species worldwide (Li et al., 2014; Yang et al., 2014; Zhao et al., 2015a,b). Genotype EbpA has been reported in only a few human cases of microsporidiosis among immunocompetent humans in the Czech Republic (Sak et al., 2011), HIV-infected individuals in Nigeria (Akinbo et al., 2012), and two children in Shanghai, China (Wang et al., 2013a), whereas genotype EbpC has been detected in humans in Vietnam, Thailand, Peru, the Czech Republic, and China (Santín and Fayer, 2011; Yang et al., 2014). These two genotypes have also been identified in nonhuman primates, pigs, dogs, cattle, sheep, goats, horses, and wastewater in China (Thellier and Breton, 2008; Qi et al., 2016; Shi et al., 2016). Recent genetic analysis using ITS and mini- and microsatellite markers showed that directed evolution of EbpC to other genotypes in swine and humans samples (Wan et al., 2016). Genotypes HLJDI and HLJD-IV were originally and uniquely identified in sika deer (Zhao et al., 2014), whereas COS-I was previously identified in sheep, dairy cattle, and Pere David's deer (Zhang et al., 2015; Li et al., 2016).

In our phylogenetic analysis, eight of the 18 novel *E. bieneusi* genotypes (JLD-I to JLD-VI, HND-I to HND-II) in this study clustered in group 1 and therefore have zoonotic potential and importance in public health. This group contains 94% of the published ITS genotypes of *E. bieneusi* and almost all the human-pathogenic genotypes (Henriques-Gil et al., 2010). The remaining 10 new genotypes were classified in the so-called “cattle-specific” group 2. Several genotypes of group 2 (such as BEB6, I, and J) have been detected in humans in recent years (Wang et al., 2013a), so some genotypes in group 2 may have limited zoonotic potential. Among the seven established genotypes detected in this study, four (BEB6, D, EbpA, and EbpC) have been reported in humans and a wide range of animals, and eight of the 18 novel genotypes clustered in group 1, with zoonotic potential, suggesting that deer may play an important role in the transmission of *E. bieneusi* genotypes among various host species, including humans.

In conclusion, the data presented in this study demonstrate that *E. bieneusi* infection is common in cervids in China, and that genotype BEB6 showed an absolute predominance in the investigated areas. The detection of four genotypes (BEB6, D, EbpA, and EbpC) known to infect humans in sika deer and red deer and the fact that eight novel genotypes (JLD-I to JLD-VI, HND-I to HND-II) belong to group 1 suggest that deer are a potential source of *E. bieneusi* infection in humans. Therefore, it is essential to investigate more fully the transmission dynamics between deer and humans in different geographic regions.

## Author contributions

Conceived and designed the experiments: LZ, RW. Performed the experiments: JH, ZZ. Analyzed the data: JH, ZZ, JZ. Contributed reagents/materials/analysis tools: YY, FJ, CN. Wrote the paper: JH, RW.

## Funding

This study was supported in part by the National Natural Science Foundation of China (313020793, 31110103901), the Key Program of the National Natural Science Foundation of China (31330079), and the Program for Science and Technology Innovation Talents in Universities of Henan Province (16HASTIT018).

### Conflict of interest statement

The authors declare that the research was conducted in the absence of any commercial or financial relationships that could be construed as a potential conflict of interest.
